# Improved Cryopreservation of Human Umbilical Vein Endothelial Cells: A Systematic Approach

**DOI:** 10.1038/srep34393

**Published:** 2016-10-06

**Authors:** A. Billal Sultani, Leah A. Marquez-Curtis, Janet A. W. Elliott, Locksley E. McGann

**Affiliations:** 1 Department of Chemical and Materials Engineering, University of Alberta, Edmonton, Alberta, Canada; 2 Department of Laboratory Medicine and Pathology, University of Alberta, Edmonton, Alberta, Canada

## Abstract

Cryopreservation of human umbilical vein endothelial cells (HUVECs) facilitated their commercial availability for use in vascular biology, tissue engineering and drug delivery research; however, the key variables in HUVEC cryopreservation have not been comprehensively studied. HUVECs are typically cryopreserved by cooling at 1 °C/min in the presence of 10% dimethyl sulfoxide (DMSO). We applied interrupted slow cooling (graded freezing) and interrupted rapid cooling with a hold time (two-step freezing) to identify where in the cooling process cryoinjury to HUVECs occurs. We found that linear cooling at 1 °C/min resulted in higher membrane integrities than linear cooling at 0.2 °C/min or nonlinear two-step freezing. DMSO addition procedures and compositions were also investigated. By combining hydroxyethyl starch with DMSO, HUVEC viability after cryopreservation was improved compared to measured viabilities of commercially available cryopreserved HUVECs and viabilities for HUVEC cryopreservation studies reported in the literature. Furthermore, HUVECs cryopreserved using our improved procedure showed high tube forming capability in a post-thaw angiogenesis assay, a standard indicator of endothelial cell function. As well as presenting superior cryopreservation procedures for HUVECs, the methods developed here can serve as a model to optimize the cryopreservation of other cells.

Human umbilical vein endothelial cells (HUVECs) have become a model system for vascular biology research since their successful culture in 1973^1^. HUVECs are used to study physiology and pathophysiology of vascular disorders^2^, biomaterials in tissue engineering^3^,^4^ and drug delivery systems^5^,^6^. Investigations and applications include: vasoregulation^7^, coagulation^8^, fibrinolysis^9^, atherosclerosis^10^, vasculogenesis and angiogenesis^11^ and as a healthy counterpart to dysfunctional endothelial cells^12^. Their availability has been facilitated through routine cryopreservation procedures^13^,^14^,^15^ that were originally designed for corneal cells^16^,^17^. Despite substantial research on HUVECs, the key variables in their cryopreservation have not been optimized.

Cell response to freeze-thaw stress is an important first step to investigate cryopreservation of cells, and the plasma membrane is of particular interest^18^. Ice excludes solutes to the unfrozen fraction^19^, thus increasing solute concentration and creating osmotic imbalance. The cells restore equilibrium either by undergoing intracellular ice formation or by becoming sufficiently dehydrated^20^. The mechanism by which intracellular ice formation occurs has been linked directly to membrane damage, with the proposition that intracellular ice is a result rather than a cause of damage^21^. On the other hand, cells can only lose water to a certain extent before it becomes lethal^22^.

Mazur developed the two-factor hypothesis of freezing injury to explain observations of optimal cooling rates^23^. Cooling cells slower than the optimal rate in the presence of ice results in cell death by excessive dehydration and solute toxicity^24^,^25^ while cooling cells faster than the optimal rate results in cell death by intracellular ice formation^21^. Many types of cells which are rapidly cooled can be saved from freezing injury by rapid thawing^26^. Cryoprotectants also mitigate slow cooling damage and enable survival of cells at lower cooling rates. Cryoprotectants can be classified based on their ability to permeate cell membranes^27^. Permeating cryoprotectants pass through cell membranes, protecting cells by increasing intracellular and extracellular osmolality^28^,^29^, depressing the freezing temperature thereby reducing the amount of ice formed^29^,^30^,^31^, and reducing the extent of cell shrinkage^28^. Dimethyl sulfoxide (DMSO) is a water-soluble permeating cryoprotectant and was first demonstrated for human and bovine red blood cells and bull spermatozoa^32^,^33^,^34^. Non-permeating cryoprotectants, which are incapable of diffusing through intact cell membranes, protect cells by increasing extracellular osmolality, causing cells to dehydrate and reducing the likelihood of intracellular ice formation and the amount of ice formed^35^,^36^,^37^. Hydroxyethyl starch (HES) was first demonstrated as a non-permeating cryoprotectant for erythrocytes^38^, and a low molecular weight HES (Pentastarch) has been used as a plasma volume expander^39^. The use of HES in clinical settings makes it an ideal cryoprotectant for human health therapeutics. A combination of DMSO and HES has been used to cryopreserve many cells, including: *i*) umbilical cord blood cells^40^, *ii*) human bone marrow^41^, *iii*) peripheral blood stem/progenitor cells^42^,^43^, *iv*) granulocytes^44^, *v*) human monocytes^45^, *vi*) canine bone marrow CD34^+^ cells^46^, and *vii*) canine pancreatic islet cells^47^, but did not lead to any improvement over using DMSO alone for platelets^48^ and in some hematopoietic stem cell studies^49^,^50^. A wide variety of concentration combinations for DMSO and HES have been recommended for different cell types^40^,^41^,^42^,^43^,^44^,^45^,^46^,^47^,^51^,^52^,^53^,^54^,^55^,^56^. A combination of DMSO and HES has not been previously considered for endothelial cells.

Cryoprotectants, although beneficial, can introduce stress to cells. Volume excursions during their addition and removal can be damaging to cell membranes^25^ and depending on concentration, cryoprotectants can be toxic which can cause greater damage than osmotic stress^57^. The degree by which cell volumes change depends on: *i*) hydraulic conductivity, a membrane characteristic used to describe water diffusion across the cell membrane^58^, *ii*) solute permeability, that describes solute diffusion across the cell membrane^59^,^60^, and *iii*) intracellular solution osmotic virial coefficients, used to describe changes in intracellular osmolality as a function of solute concentration^61^. To maximize cryoprotection and minimize toxicity, lower concentrations of cryoprotectants, shorter exposure times and lower temperatures are favorable^22^,^62^.

HUVEC cryopreservation has been studied using intact umbilical veins^63^, the HUVEC cell line ECV304^15^, and HUVEC suspensions^13^,^14^,^64^,^65^,^66^. Cryopreservation of HUVEC suspensions resulted in a wide range of cell recovery due to sample variability and technician differences^13^. Cooling was performed at a rate of 1 °C/min in 10% DMSO in defined media (CPTes) followed by storage for 7 to 36 days in the liquid nitrogen vapour phase. Upon thawing, 66 ± 5% viable cells were recovered (mean ± standard deviation, n = 31, range 32% to 88%) using the trypan blue exclusion assay^13^. Good manufacturing practice in the cryopreservation of HUVECs in 10% DMSO and 18% human serum albumin, cooled nominally at 1 °C/min to –80 °C and stored in the liquid nitrogen vapour phase resulted in post-thaw viabilities of 66.3 ± 4.4% (7 days in liquid nitrogen vapour) and 69.2 ± 2.1% (1 year in liquid nitrogen vapour)^64^.

Interrupted cooling protocols enable optimization of cryopreservation procedures by delineating the cell damage that occurs upon cooling to intermediate sub-zero temperatures from the damage that is evident after plunging into liquid nitrogen^30^,^67^,^68^,^69^. Viability is assessed after cooling to and thawing from intermediate sub-zero temperatures (direct thaw) or after cooling to and thawing from liquid nitrogen (plunge thaw). A schematic of the procedure is shown in Fig. 1a. The two interrupted cooling protocols of interest, two-step freezing (rapid cooling with hold time)^29^,^68^ and graded freezing (slow cooling)^26^,^67^,^69^, are shown as temperature profiles in Fig. 1b,d, respectively. HUVECs have been previously studied in the absence of cryoprotectants using both protocols where it has been found that slow cooling results in higher viabilities than rapid cooling^66^.

Because HUVECs are commonly used as a model system for the study of angiogenesis, one of the well-established assays to demonstrate the function of HUVECs is tube formation in the reconstituted basement membrane Matrigel^70^. Angiogenesis, the development of new blood vessels from pre-existing ones, is essential in normal tissue development and many pathological conditions, and is mediated primarily by endothelial cells^71^. The tube formation assay is a simple, well-established assay that recapitulates *in vitro* the multiple steps that take place during angiogenesis. These include: disruption of the basement membrane, migration of endothelial cells, and the proliferation and differentiation into capillaries, via adhesion molecule signaling and extracellular matrix remodeling, which can be observed as three-dimensional capillary-like tubular structures by microscopy^72^,^73^.

The primary objective of this work was to study cryoinjury to HUVECs by applying interrupted cooling protocols which can identify key variables for optimizing HUVEC cryopreservation. Figure 2 is a schematic diagram of the experimental design to systematically investigate the effects of: *i*) absence or presence of 10% DMSO; *ii*) cooling profiles, *iii*) DMSO addition procedures, *iv*) cryoprotectant compositions (DMSO plus HES), and *v*) plunge temperatures. Because 10% DMSO is the most common cryoprotectant used, we first compared post-thaw membrane integrities of HUVECs subjected to graded freezing *vs*. two-step freezing in the absence or presence of 10% DMSO. Next, we investigated the effect of two cooling rates (0.2 °C/min or 1 °C/min) on graded freezing. As cryoprotectants can impose an osmotic stress resulting in excessive cell shrinkage during addition and cell expansion during removal^25^,^74^, particularly for permeating cryoprotectants, graded freezing using a 1 °C/min cooling rate was used to compare three DMSO addition procedures. To investigate the effect of additional cryoprotectants, the DMSO addition procedure and the interrupted cooling protocol that resulted in the highest membrane integrity were used. Four cryoprotectant solutions were compared: *i*) 20% DMSO, *ii*) 10% DMSO plus 5% HES, *iii*) 10% DMSO plus 8% HES, and *iv*) 10% DMSO plus 10% HES. Finally, the effect of using a lower concentration of DMSO was evaluated by comparing four cryoprotectant solutions: *i*) 7% DMSO plus 7% HES, *ii*) 7% DMSO plus 6% HES, *iii*) 5% DMSO plus 6% HES and *iv*) 3% DMSO plus 6% HES.

The second objective was to compare the membrane integrity of HUVECs following the best cryopreservation procedure identified in this work to the viabilities reported in the literature^14^,^64^, and the viability of HUVECs as supplied commercially. The third objective was to demonstrate that HUVECs cryopreserved using the best protocol identified in this work also were functional based on a post-thaw angiogenesis assay.

## Results

### HUVEC Controls in the Absence or Presence of DMSO

Table 1 shows that, in the absence of cryoprotectant, the membrane integrity measured prior to performing interrupted cooling experiments and membrane integrity of HUVECS after approximately 1 hour at 0 °C remained high (p = 0.17), but was negligible after direct plunge from 0 °C into liquid nitrogen. Three DMSO addition procedures were examined for controls using a final concentration of 10% DMSO. Also, one control experiment was performed using 20% DMSO. No effect on membrane integrity was observed prior to interrupted cooling experiments as a result of DMSO addition procedure. Membrane integrities were not significantly different in the presence or absence of 10% DMSO (p > 0.3). However, in the presence of 20% DMSO, membrane integrity decreased to 84.6 ± 0.4% (p = 0.001). After 1 hour exposure to 10% DMSO at 0 °C, regardless of addition procedure, no effect on membrane integrity was observed (p > 0.6). In the presence of 20% DMSO, membrane integrity was lower at 77.1 ± 2.4% (p = 0.008), demonstrating that 1 hour exposure to 20% DMSO at 0 °C may be damaging to HUVECs. In all cases, the measured membrane integrity after plunging HUVECs into liquid nitrogen from 0 °C without any controlled cooling was very low, less than 5%.

### Effect of Cooling Rate in the Absence or Presence of DMSO

Figure 3a shows direct thaw and plunge thaw membrane integrities in the absence of cryoprotectant after: *i*) two-step freezing using a 3-minute hold time, *ii*) graded freezing using a 0.2 °C/min cooling rate, and *iii*) graded freezing using a 1 °C/min cooling rate. In all cases, membrane integrity after direct thaw decreased gradually as temperature decreased; however, direct thaw from −12 °C and −15 °C showed that two-step freezing resulted in significantly lower membrane integrities (p < 0.008). Graded freezing using a 1 °C/min cooling rate resulted in the highest membrane integrities (p < 0.02). Membrane integrities after plunge thaw were very low (<2%) in all cases.

Figure 3b shows results of two-step freezing and graded freezing using a 1 °C/min or 0.2 °C/min cooling rate in the presence of 10% DMSO. The membrane integrity of HUVECs subjected to graded freezing using a 1 °C/min or 0.2 °C/min cooling rate remained high after direct thaw, ranging from 92.5 ± 0.3% to 90.7 ± 0.7%. Moreover, membrane integrities after direct thaw were significantly higher after graded freezing than two-step freezing for experimental temperatures in the range of −20 °C to −40 °C (p < 0.02). Membrane integrities after plunge thaw were also higher after graded freezing than two-step freezing when plunging from −25 °C or lower (p < 0.05). Membrane integrities were higher after plunge thaw from graded freezing using a 1 °C/min cooling rate compared to using a 0.2 °C/min cooling rate for experimental temperatures in the range of −20 °C to −40 °C (p < 0.05). The highest membrane integrity attained after plunge thaw was 67.4 ± 1.9% using a 1 °C/min cooling rate to −35 °C.

### Effect of DMSO Addition Procedures

To compare the effect of DMSO addition procedures, graded freezing was performed using a 1 °C/min cooling rate at a final concentration of 10% DMSO. Figure 4 shows membrane integrity results for three different DMSO addition procedures at 0 °C: *i*) HUVECs exposed to 10% DMSO for 15 minutes, *ii*) HUVECs exposed to 10% DMSO for 30 minutes, and *iii*) a multi-step DMSO addition procedure where HUVECs are initially exposed to 3% DMSO for 10 minutes followed by 10% DMSO for 20 minutes. This third procedure was proposed by Pegg^15^, and modified in this work by eliminating the centrifugation step. There were no significant differences in membrane integrities among plunge thaw samples except at −30 °C between 15 minute and 30 minute exposure (p = 0.03) and at −5 °C between 15 minute exposure and multi-step procedure (p = 0.003). Membrane integrity remained high after direct thaw from all experimental temperatures. Since there was no significant difference in membrane integrities observed between the different DMSO addition procedures used with graded freezing, adding cryoprotectant using a 15 minute exposure at 0 °C was used for all subsequent experiments.

### Graded Freezing Using Increased Cryoprotectants

Figure 5 shows membrane integrity results after graded freezing using a 1 °C/min cooling rate in the presence of: *i*) 10% DMSO, *ii*) 10% DMSO plus 5% HES, *iii*) 10% DMSO plus 8% HES, and *iv*) 20% DMSO. Except between 10% DMSO and 20% DMSO at −20 °C (p = 0.03) and −30 °C (p = 0.04), the membrane integrities after direct thaw were not significantly different comparing all four graded freezing experiments (p ≥ 0.05). After plunge thaw, the membrane integrity was significantly higher for 10% DMSO compared to 20% DMSO at −25, −35 and −45 °C (p < 0.05); however, it must be noted from the flow cytometry membrane integrity data that the background light scatter was much higher in the presence of 20% DMSO, and as well the compensation was reduced from 32.0% to 29.0% in the presence of 20% DMSO due to a decrease in fluorescence from membrane-intact cells. In general, the membrane integrities were significantly higher for 10% DMSO plus 5% HES after plunge thaw compared to 10% DMSO alone except at −15, −20, and −25 °C (p ≥ 0.2). The membrane integrities were higher still for 10% DMSO plus 8% HES after plunge thaw except at −25 °C (p = 0.1) A clear optimum temperature was not determined for plunge thaw using 10% DMSO plus 8% HES as membrane integrity continued to increase with decreasing experimental plunge temperature, with the highest membrane integrity of 83.6 ± 1.6% after plunge thaw from −45 °C.

### Graded Freezing using Reduced Concentrations of DMSO and HES

We next investigated whether the high membrane integrity would be retained if we reduce the concentration of cryoprotectants. Two different combinations of DMSO plus HES were compared by performing graded freezing using a 1 °C/min cooling rate: *i*) 5% DMSO plus 6% HES, and *ii*) 3% DMSO plus 6% HES. Figure 6 shows membrane integrity measured after direct thaw and after plunge thaw from four experimental temperatures: −15 °C, −25 °C, −35 °C and −45 °C. Membrane integrity remained high after direct thaw from all experimental temperatures in the presence of 5% DMSO plus 6% HES; however membrane integrity decreased significantly after direct thaw from −35 °C (p = 0.017) and −45 °C (p = 0.001) in the presence of 3% DMSO plus 6% HES. After plunge thaw, membrane integrity reached a maximum of 87.7 ± 0.8% at −35 °C and remained high as the temperature decreased to −45 °C in the presence of 5% DMSO plus 6% HES (p = 0.23). In the presence of 3% DMSO plus 6% HES, membrane integrity reached a maximum of 79.3 ± 2.1% after plunge thaw from −35 °C; however due to lower membrane integrities after direct thaw from −45 °C, membrane integrity was significantly lower after plunge thaw from −45 °C (p = 0.003).

### Maximum Viabilities for Combinations of DMSO plus HES

In addition to the concentrations of DMSO and HES previously described, other combinations were tested: *i*) 10% DMSO plus 10% HES, *ii*) 7% DMSO plus 7% HES, and *iii*) 7% DMSO plus 6% HES. Table 2 summarizes the maximum membrane integrities attained after incubating HUVECs with cryoprotectants for 15 minutes on ice and performing graded freezing using a 1 °C/min cooling rate. Using 10% DMSO plus 10% HES significantly lowered the maximum membrane integrity after plunge thaw compared to using 10% DMSO plus 8% HES (p = 0.042). Using 7% DMSO plus 7% HES, the maximum membrane integrity is similar to using 10% DMSO plus 8% HES (p = 0.42) despite the lower concentration of DMSO. It appears that there is a range of concentrations of HES between 5% and 10% that results in higher membrane integrities compared to 10% DMSO alone. The highest membrane integrity attained after plunge thaw was 87.7 ± 0.8% in the presence of 5% DMSO plus 6% HES.

### Assessing Tube Formation for the Protocol that Yielded the Highest Membrane Integrity

The ability of HUVECs to promote angiogenesis *in vitro* after being subjected to graded freezing using a 1 °C/min cooling rate in the presence of 5% DMSO plus 6% HES was evaluated using a tube formation assay. Figure 7a shows representative phase contrast images of tube formation by HUVECs rapidly thawed from various sub-zero plunge temperatures and plated on Matrigel. The degree of formation of a network of capillary-like tubules increased as the temperature at which they were plunged into liquid nitrogen decreased. The tube length was used to quantify the extent of tube formation and the percent membrane integrity was used to measure the population of membrane-intact (viable) cells. When the tube length and membrane integrity were both normalized relative to fresh, unfrozen (control) cells, there was no significant difference between the post-thaw membrane integrity and tube formation in HUVECs plunged in liquid nitrogen from various sub-zero temperatures (Fig. 7b). HUVECs cryopreserved using the best cryopreservation procedure had high normalized membrane integrity (94.0 ± 0.9%) and a large extent of normalized tube formation (85.8 ± 6.2%) relative to that of fresh HUVECs.

## Discussion

**Figure 1:**
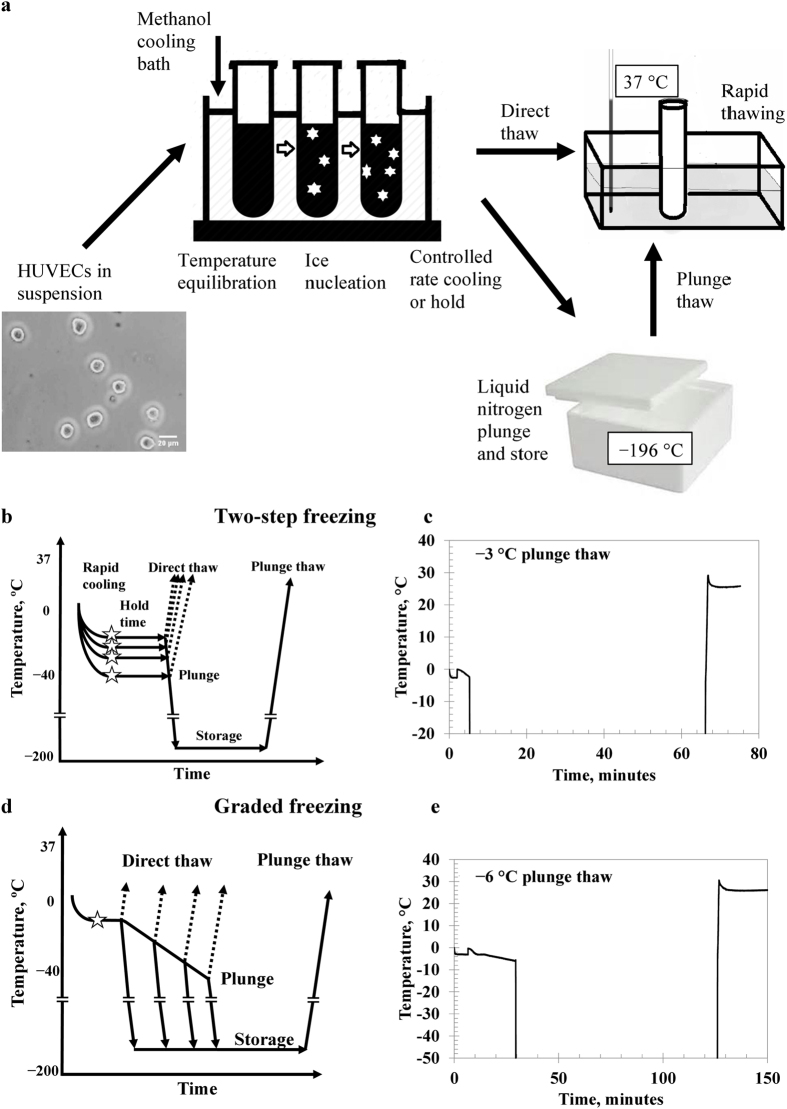
Schematic diagram of the experimental set-up and temperature profiles for two-step freezing and graded freezing. **(a**) HUVECs in suspension were subjected to interrupted cooling in a methanol bath and either directly thawed, or plunged and stored in liquid nitrogen before thawing in a 37 °C water bath. **(b**) Schematic diagram of two-step freezing which involves: (*i*) rapid cooling to intermediate sub-zero temperatures (hold temperatures), (*ii*) induced ice formation (☆) using liquid nitrogen-cooled forceps, (*iii*) holding for 3 minutes at intermediate sub-zero temperatures (*iv*) rapid plunge into liquid nitrogen, (*v*) storage in liquid nitrogen and (*vi*) rapid thawing. Steps (*i*), (*ii*), (*iii*) and (*vi*) are performed for direct thaw, and all steps are performed for plunge thaw. **(c**) Representative temperature trace of two-step freezing for –3 °C plunge thaw. **(d**) Schematic diagram of graded freezing which involves: (*i*) induced ice formation (☆) using liquid nitrogen-cooled forceps, (*ii*) holding for 3 minutes at the first experimental temperature, (*iii*) controlled cooling at 1 °C/min or 0.2 °C/min to intermediate sub-zero temperatures (experimental temperatures), (*iv*) rapid plunge into liquid nitrogen, (*v*) storage in liquid nitrogen and (*vi*) rapid thawing. Steps (*i*), (*ii*), (*iii*) and (*vi*) are performed for direct thaw, and all steps are performed for plunge thaw. **(e**) Representative temperature trace of graded freezing at 0.2 °C/min for −6 °C plunge thaw.

**Figure 2:**
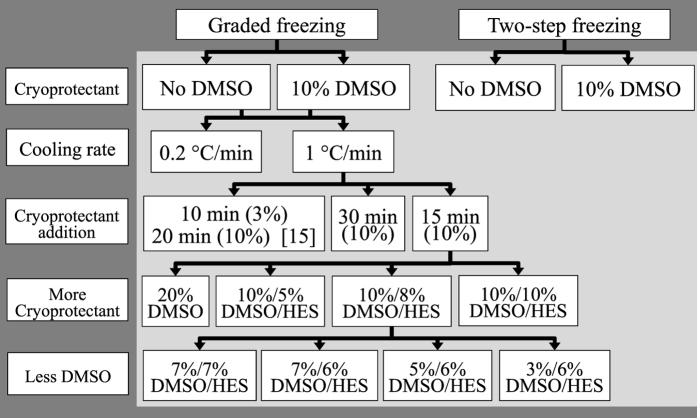
Experimental design for determining key variables to optimize the cryopreservation of HUVECs. The cells were first subjected to graded freezing *vs.* two-step freezing in the absence or presence of 10% DMSO. Next, the effect of two cooling rates (0.2 °C/min or 1 °C/min) on graded freezing was examined. Then, graded freezing using a 1 °C/min cooling rate was used to compare three DMSO addition procedures (see Fig. 4 caption for details). To investigate the effect of additional cryoprotectants, the DMSO addition procedure and the interrupted cooling protocol that resulted in the highest membrane integrity were used. Four cryoprotectant solutions were compared: (*i*) 20% DMSO, (*ii*) 10% DMSO plus 5% HES, (*iii*) 10% DMSO plus 8% HES, and (*iv*) 10% DMSO plus 10% HES. Finally, the effect of using a lower concentration of DMSO was evaluated by comparing four cryoprotectant solutions: (*i*) 7% DMSO plus 7% HES, (*ii*) 7% DMSO plus 6% HES, (*iii*) 5% DMSO plus 6% HES and (*iv*) 3% DMSO plus 6% HES.

This work had three objectives. First, HUVEC cryoinjury was studied by applying interrupted cooling protocols (Fig. 1) to identify key variables to optimize HUVEC cryopreservation (Fig. 2). Second, the membrane integrities of HUVECS after the best cryopreservation procedure in this work were compared with membrane integrities of cryopreserved HUVECS reported in the literature and cryopreserved HUVECs as supplied. Third, membrane integrity of HUVECs based on an assay developed for this work was compared to their functionality based on a post-thaw angiogenesis assay.

**Figure 3:**
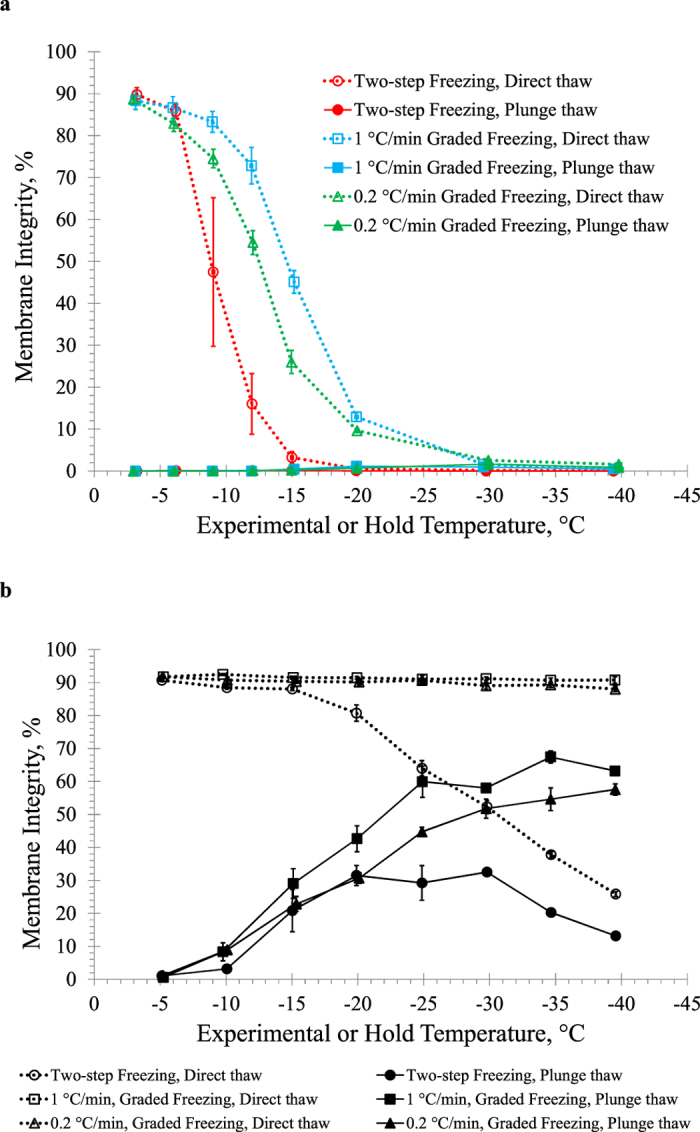
Effect of cooling rate on interrupted cooling of HUVECs (**a**) in the absence of cryoprotectant, or (**b**) in the presence of 10% DMSO. The cells were either rapidly cooled to an intermediate subzero temperature and held at that temperature (two-step freezing), or slowly cooled at 1 °C/min or 0.2 °C/min to intermediate sub-zero temperatures, before direct thaw or plunge thaw. Three independent experiments were carried out and the mean membrane integrity was calculated for each experimental temperature. Error bars represent standard error of the mean.

It is known that rapidly cooling cells using interrupted rapid cooling with hold time helps to identify membrane damage resulting from intracellular ice formation^68^ and interrupted slow cooling in the presence of ice helps to identify membrane damage from solute effects^69^. In the absence of cryoprotectants, we observed higher membrane integrities after direct thaw from graded freezing using a 1 °C/min cooling rate compared to graded freezing using a 0.2 °C/min cooling rate or two-step freezing. In the absence of cryoprotectants (Fig. 3a), a large amount of cell damage occurred and membrane integrities were very low after plunge thaw.

We next investigated the effect of different cooling protocols in the presence of 10% DMSO (Fig. 3b). Membrane integrity was much higher than in the absence of cryoprotectant. It is known that DMSO has a high solubility in water at low sub-zero temperatures and reduces ice formation, making it effective as a permeating cryoprotectant^33^,^34^. DMSO protects cells from freezing injury by increasing the intracellular and extracellular osmolality^28^,^29^, which reduces the amount of ice formed^29^,^30^,^31^,^75^ and cell shrinkage^28^ at sub-zero temperatures. For interrupted cooling protocols in the presence of 10% DMSO, the best cooling profile was graded freezing using a 1 °C/min cooling rate. Graded freezing allows more time for cell dehydration and less damage from supercooling effects upon plunge into liquid nitrogen than two-step freezing using a 3-minute hold time; therefore, slow cooling results in higher membrane integrities after plunge thaw from −30 °C from reduced supercooling effects. In the presence of 10% DMSO, the interrupted cooling protocol direct thaw results demonstrate that HUVECs are protected from supercooling and solute effects to as low as −15 °C. For two-step cooling, rapid cooling to temperatures lower than −15 °C would be beneficial due to higher osmolalities during the 3-minute hold time which would allow cells to dehydrate more and reduce supercooling effects; however 10% DMSO and the 3-minute hold time is insufficient to completely protect cells from rapid cooling below −15 °C.

**Figure 4:**
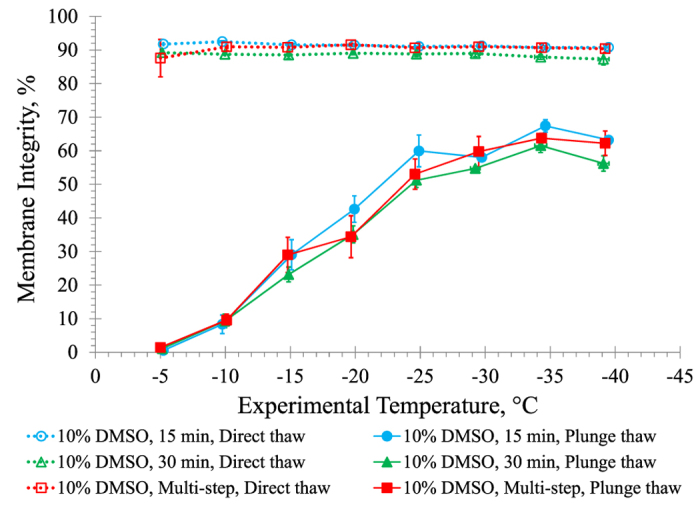
Comparison of the effect of DMSO addition procedures on graded freezing using a 1 °C/min cooling rate. The three different DMSO procedures tested were: (*i*) adding a 20% DMSO solution to the HUVEC suspension to a final concentration of 10% DMSO with 15 minute exposure at 0 °C; (*ii*) adding a 20% DMSO solution to the HUVEC suspension to a final concentration of 10% DMSO with 30-minute exposure at 0 °C; and (*iii*) adding a 20% DMSO solution to the HUVEC suspension to an initial concentration of 3% DMSO followed by a 10-minute exposure at 0 °C, and then adding more of the 20% DMSO solution to a final concentration of 10% DMSO followed by a 20-minute exposure at 0 °C^15^. Three independent experiments were carried out and the mean membrane integrity was calculated for each experimental temperature. Error bars represent standard error of the mean.

Next, we examined whether the osmotic stress experienced by the cells may be mitigated by DMSO addition procedures (Fig. 4). No differences in membrane integrities were observed after exposing HUVECs to 10% DMSO for either 15 minutes or 30 minutes at 0 °C followed by graded freezing at 1 °C/min, suggesting that 15 minutes is sufficient to allow 10% DMSO to fully permeate HUVECs. Regardless of the procedure to add 10% DMSO, as HUVECs were cooled to lower temperatures, more HUVECs survived plunge thaw. As shown here the multi-step addition procedure proposed by Pegg^15^ is not necessary because 10% DMSO poses negligible osmotic stress during addition at 0 °C. There was also no effect on membrane integrity from 1 hour exposure to 10% DMSO at 0 °C. Thus, the DMSO addition procedure was not identified as a variable requiring optimization.

**Figure 5:**
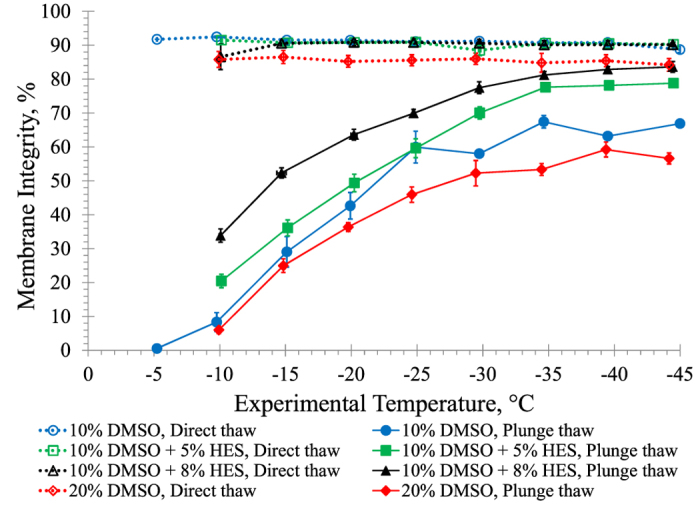
Membrane integrities of HUVECs after graded freezing using a 1 °C/min cooling rate to various sub-zero temperatures in the presence of 10% DMSO, 20% DMSO, 10% DMSO + 5% HES, or 10% DMSO + 8% HES. Three independent experiments were carried out and the mean membrane integrity was calculated for each experimental temperature. Error bars represent standard error of the mean.

There was no benefit from using 20% DMSO compared to 10% DMSO during graded freezing at a 1 °C/min cooling rate after a 15 minute exposure to cryoprotectant at 0 °C (Fig. 5). It is known that DMSO can be beneficial^32^ but DMSO is also known to be toxic at high concentrations^57^. Since no improvement in viability was observed using 20% DMSO compared to 10% DMSO, there could be a balance between the benefit from higher osmolality and the negative effect from DMSO toxicity.

Next, the non-permeating cryoprotectant HES was combined with the permeating cryoprotectant DMSO. HES is known to increase the extracellular osmolality which has many advantages during freezing, including: *i*) depressing the freezing temperature, *ii*) reducing the amount of ice formed at a given temperature resulting in a lower salt concentration at a given temperature, *iii*) reducing cell volume, and *iv*) decreasing the supercooling effects^35^,^36^,^37^. It was observed that the higher the concentration of HES, the higher the viability after plunge thaw; however this was only observed up to 8% HES in the presence of 10% DMSO. Using 10% DMSO plus 10% HES resulted in lower viabilities, demonstrating that there is an optimum concentration of HES in the presence of 10% DMSO. The higher concentrations of HES in the presence of 10% DMSO may cause excessive dehydration, where the water content may be insufficient and the cell could shrink beyond a minimum tolerable cell volume^22^.

**Figure 6:**
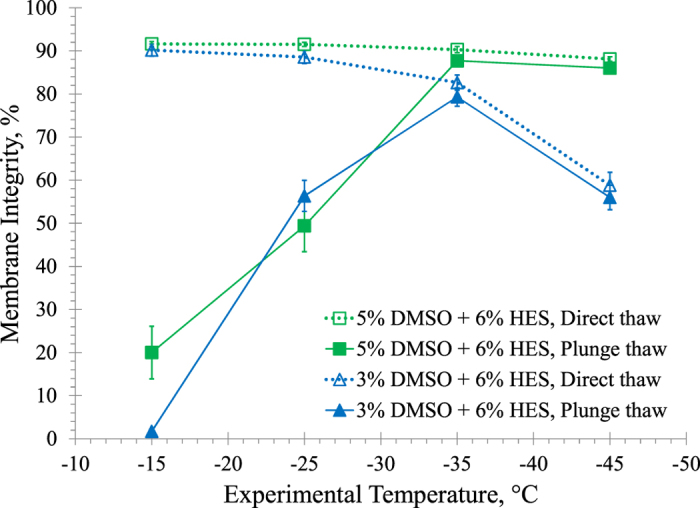
Membrane integrities of HUVECs after graded freezing using a 1 °C/min cooling rate to various sub-zero temperatures in the presence of 5% DMSO + 6% HES and 3% DMSO + 6% HES. Three independent experiments were carried out and the mean membrane integrity was calculated for each experimental temperature. Error bars represent standard error of the mean.

**Figure 7:**
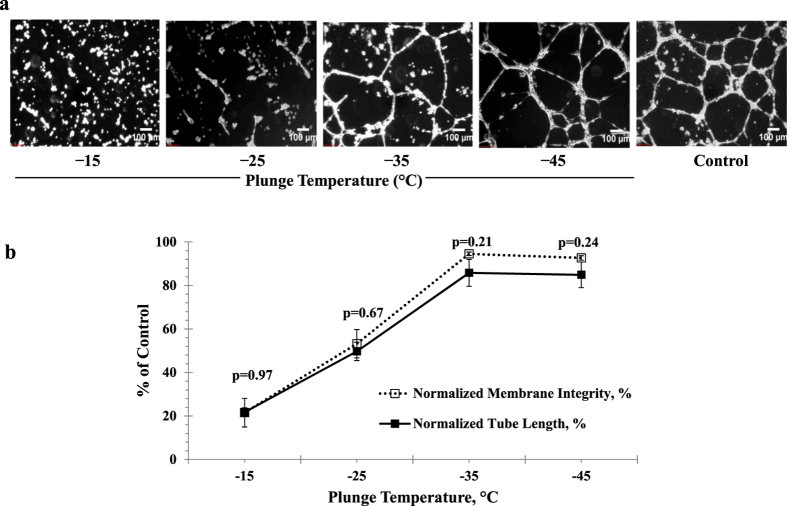
(**a**) Representative phase contrast images of tube formation, and (**b**) membrane integrity and tube lengths normalized against control cells. HUVECs were subjected to graded freezing at 1 °C/min cooling rate in the presence of 5% DMSO and 6% HES, cooled to various sub-zero temperatures, plunged into liquid nitrogen, rapidly thawed and plated on Matrigel. Images were acquired at 40X magnification. The total tube length in the cryopreserved samples was quantified (in pixels) using the NIH ImageJ software with the Angiogenesis Analyzer plugin and normalized against fresh, unfrozen (control) cells. Likewise, the membrane integrity was normalized against unfrozen (control) cells. P-values indicate that normalized membrane integrity and tube-forming ability are not significantly different for all plunge temperatures tested.

In an attempt to decrease the potential negative effects of cryoprotectants, the concentrations of DMSO and HES were reduced. Using 7% DMSO plus 7% HES, the maximum membrane integrity is similar to using 10% DMSO plus 8% HES and the maximum membrane integrity is higher than using 10% DMSO plus 10% HES. There appears to be an optimum concentration of HES with 10% DMSO which is between 5% HES and 10% HES. The highest membrane integrity in this work was attained in the presence of 5% DMSO plus 6% HES. However, decreasing the concentration of DMSO from 5% to 3% in the presence of 6% HES (Fig. 6) decreased the maximum membrane integrity. Decreasing DMSO concentration to 3% may cause an increased amount of ice present at a given sub-zero temperature, increasing exposure to higher salt concentrations and increasing supercooling effects^66^.

For hematopoietic stem cells, using 5% DMSO plus 6% HES resulted in quicker white blood cell count recovery in patients compared to cryopreservation using 10% DMSO alone^43^; other studies showed no difference^49^,^50^. Platelet recovery was lower after cryopreservation using 5% DMSO plus 6% HES compared to 6% DMSO alone^48^. Other combinations of DMSO and HES have been studied. For example, cryopreservation of peripheral blood stem cells showed that using 5% DMSO plus 3% HES resulted in higher viabilities than using 10% DMSO^51^. For rat mesenchymal stem cells, 5% DMSO plus 5% HES was recommended, although 8% DMSO plus 2% HES showed the highest viability^52^. Umbilical cord mesenchymal cells were successfully cryopreserved using a 10% DMSO plus 20% HES solution^53^. Cryopreservation of rat granulocytes was optimal using 10% DMSO plus 5% HES^54^, while 5% DMSO plus 4% HES resulted in a viability of 71.2% in human pancreatic islets^55^. Adding 5% ethylene glycol to 5% DMSO plus 6% HES improved the viability of human pluripotent stem cells to over 80%^56^. In this work, we are the first to report that cryopreserving HUVECS in the presence of 5% DMSO plus 6% HES resulted in the highest membrane integrity.

For the second objective, the best cryopreservation procedure in this work was determined to be cooling at 1 °C/min in the presence of 5% DMSO plus 6% HES to −35 °C and then storing in liquid nitrogen, yielding a maximum membrane integrity of 87.7 ± 0.8% after plunge thaw which was equivalent to 94.0 ± 0.9% when normalized against fresh, unfrozen control cells. This is about 30% higher than the membrane integrity of 64.8 ± 2.2% we measured (N = 6) for the supplier-provided HUVECs that were cryopreserved using 10% DMSO. It is also higher than the viability reported in the literature (69.2% ± 2.3%) for the standard good manufacturing practices protocol (cooling nominally at 1 °C/min in the presence of 18% human serum albumin and 10% DMSO and storing in the liquid nitrogen vapour phase)^64^. Our procedure uses low serum (only 2% fetal bovine serum (FBS) as compared with the 10% to 90% serum used in current protocols), and takes less time than slow cooling all the way to −80 °C.

The third objective was to compare HUVEC membrane integrity based on an assay developed for this work with HUVEC functionality based on a post-thaw angiogenesis assay. Cell membrane integrity assessment post-cryopreservation is important because the plasma membrane has been considered to be one of the primary sites of cryoinjury^76^,^77^. However, because cells can undergo biological changes during the freeze/thaw process, it is important to evaluate not only their membrane integrity but also their function following cryopreservation. The tube formation assay is the most appropriate *in vitro* functional assay for HUVECs because it incorporates the multiple processes that occur during angiogenesis including cell proliferation, signaling through adhesion molecules such as CD31^78^, and participation of extracellular matrix proteins such as laminin and collagen type IV^73^. Recently, we showed a strong correlation between total tube length and the number of functional HUVECs^79^. Here we demonstrate that HUVECs incubated for 15 minutes on ice in the presence of 5% DMSO plus 6% HES, and subjected to graded freezing using a 1 °C/min cooling rate and −35 °C plunge thaw yielded cells of the highest membrane integrity and with tube forming ability similar to that of fresh unfrozen cells. In our previous work, we have shown that for HUVECs subjected to graded freezing at 1 °C/min in the presence of 10% DMSO, the extent of tube formation correlated with the number of membrane-intact cells at the higher membrane integrities (*i.e.*, lower plunge temperatures)^79^. In this work, graded freezing at 1 °C/min in the presence of 5% DMSO plus 6% HES resulted in membrane integrities and tube forming abilities that were not significantly different at all plunge temperatures tested. HES may have an additional protective ability in the presence of DMSO that extends to preserving functionality as well as membrane integrity. The ability to recover cells of high viability and functionality after cryopreservation will be useful in research investigations and tissue engineering applications that employ HUVECs.

In conclusion, interrupted cooling protocols facilitated the identification of cryoinjury allowing us to systematically optimize the cryopreservation procedure. The presence of cryoprotectants, cooling rates, cryoprotectant combinations and concentrations all affected the viability of cryopreserved HUVECs. Only the DMSO addition procedure did not have a significant effect. The best procedure for cryopreserving HUVECS, identified in this work, was to cool HUVECs at 1 °C/min in the presence of 5% DMSO plus 6% HES to −35 °C, and then to plunge into liquid nitrogen and store, followed by rapid warming in a 37 °C water bath. The membrane integrity of HUVECs using this best cryopreservation procedure (87.7 ± 0.8%; 94.0 ± 0.9% when normalized against fresh, unfrozen control) is higher than the viability of 66.3 ± 4.4% (7 days in liquid nitrogen vapour) and 69.2 ± 2.3% (1 year in liquid nitrogen vapour) reported in the literature using a standard good manufacturing practices protocol^64^. Also, the best cryopreservation procedure reported herein results in a higher membrane integrity than the 64.8 ± 2.2% we measured for the cryopreserved HUVECs as supplied.

The HUVECs cryopreserved using our optimized procedure exhibited functionality (tube forming ability) that is not statistically significantly different from the number of membrane-intact HUVECs. The procedural detail provided in this work and further described in a thesis^80^ is appropriate to ensure high reproducibility of results, and can be used to optimize the cryopreservation of other types of cells. However, although interrupted cooling protocols provide a means to determine a range of optimal cooling conditions, it must be emphasized that the responses are cell-type specific, since cell responses are governed by cell-specific characteristics such as hydraulic conductivity^58^, membrane solute permeability^59^,^60^ and intracellular solution osmotic behavior^61^. This work provides the understanding and framework necessary to efficiently design protocols in order to achieve remarkable recovery of membrane-intact and functional cells after cryopreservation.

## Methods

### HUVEC Cultures

HUVECs (Lonza Group Ltd., Walkersville, MD, USA) were purchased as pooled primary cells frozen after the first sub-culture. They were supplied in a cryopreservation medium containing endothelial growth medium (EGM) with 10% FBS and 10% DMSO. HUVECs were shipped (Cedarlane, Burlington, ON, Canada) in a polystyrene container with dry ice and immediately stored in liquid nitrogen until required.

HUVECs were cultured according to manufacturer’s instructions^81^ except that the cells were: *i*) cultured in the absence of antibiotics, *ii*) passaged when they reached 50% to 80% surface coverage, *iii*) centrifuged at 200g for 5 minutes in an Eppendorf 5810R tabletop centrifuge (Eppendorf AG, Hamburg, Germany), *iv*) prepared for interrupted cooling experiments using a cell suspension in EGM at a concentration of approximately 1 × 10^6^ to 2 × 10^6 ^cells/mL, and *v*) placed on wet ice at 0 °C for 2–4 hours prior to experiments. HUVECs were used up to15 population doublings which corresponded to sub-cultures of up to passage 6.

### Temperature Measurements

A T-type thermocouple and OMB-DAQ-55 data acquisition module (OMEGA Engineering Inc., Stamford, CT, USA) were used to measure temperature. A methanol bath (FTS Systems, Stone Ridge, NY, USA) was used to control the cooling bath temperature and cooling rates in all interrupted cooling experiments. Temperature referencing at 0 °C was performed by placing thermocouples in the water portion of an ice–water bath and recording the measurement of each thermocouple. A thermocouple was then inserted in a borosilicate glass culture tube (VWR, Edmonton, AB, Canada) containing 200 μL of EGM that was placed in the methanol bath to act as a proxy for temperature measurement of HUVEC suspensions.

### Membrane Integrity Measurement by Flow Cytometry

The dual fluorescent stain (SYTOEB) containing SYTO 13 (Molecular Probes, Eugene, OR, USA) and ethidium bromide (EB) (Sigma-Aldrich, Mississauga, ON, Canada) was used to assess HUVEC membrane integrity by flow cytometry. To prepare the stock SYTOEB staining solution, 5 mM of SYTO 13 and 26 mM of ethidium bromide (EB) were combined in water. HUVECs were incubated with 11.4 μM SYTO 13 and 92.2 μM EB in the dark for 10 minutes at room temperature before membrane integrity measurement. The hazards inherent to using EB made it prudent to consider an alternative stain, propidium iodide (PI) (Life Technologies, Burlington, ON, Canada). The SYTOPI stock solution was prepared by combining 5 mM of SYTO 13 and 1.5 mM of PI in water. HUVECs were incubated with 11.4 μM SYTO 13 and 67.8 μM PI in the dark for 10 minutes at room temperature before membrane integrity measurement by flow cytometry. As shown in the Supplementary Material, the two stains (SYTOEB and SYTOPI) yielded comparable results. Which of these stains (SYTOEB or SYTOPI) was used in each experimental run is detailed in a thesis^80^ and summarized in Supplementary Table S1. Safety can further be improved by using an alternative stain to propidium iodide such as GelRed.

To measure the membrane integrity of HUVECs from the supplier, 400 μL of HUVEC suspension was taken directly from the 1.5-mL cryovials from several lots (N = 6) after thawing. The HUVEC suspension was transferred to a flow cytometer tube and assessed for membrane integrity using either SYTOEB or SYTOPI.

An Epics XL-MCL flow cytometer (Beckman Coulter Inc., Pasadena, CA, USA) with a 488-nm laser was used for flow cytometry. Forward light scatter (FS) and side light scatter (SS) sensors detected laser light scatter and the fluorescent light (FL) sensors detected light in the 200 nm to 800 nm spectral range. Laser light scatter was filtered for the FS and SS sensors by means of a 488-nm dichroic filter and laser light scatter was blocked from the FL sensors by a 488-nm laser-blocking filter. Green fluorescence emission was separated from other light using a 505-nm to 545-nm dichroic filter for the FL1 sensor. Red fluorescence emission was separated from other light using a 605-nm to 635-nm dichroic filter for the FL3 sensor. The flow cytometry measurement of membrane integrity is illustrated in a figure in the Supplementary Material. Four populations were identified in HUVECs stained with either SYTOEB or SYTOPI: *i*) membrane-intact HUVECs (green) in the Syto quadrant, *ii*) membrane-damaged HUVECs (red) in the EB or PI quadrant, *iii*) partially membrane-damaged HUVECs (doubly-stained, blue) in the Dbl quadrant, and *iv*) background light scatter in the Bkgd quadrant. To calculate percent membrane integrity (MI), equation (1) was used, where membrane-intact cells were counted from the Syto quadrant and membrane-damaged cells were counted from the EB or PI and doubly-stained quadrants.


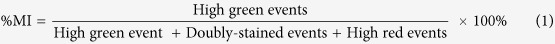


### HUVEC Controls in the Presence and Absence of DMSO

In the absence of cryoprotectants, HUVEC membrane integrity was measured prior to performing interrupted cooling experiments and after approximately 1 hour exposure at 0 °C. Also, HUVEC membrane integrity was measured for HUVECs plunged into liquid nitrogen directly from 0 °C. In the presence of DMSO, HUVEC membrane integrity was measured after DMSO addition and after approximately 1 hour exposure to DMSO at 0 °C. For these no-cooling controls, three DMSO addition procedures were examined, two direct addition procedures and one multi-step addition procedure^15^; therefore membrane integrity was measured after exposure to 10% DMSO at 0 °C for: *i*) 15 minutes, *ii*) 30 minutes, or *iii*) 10 minutes in the presence of 3% DMSO and 20 minutes in the presence of 10% DMSO^15^. Also in the presence of DMSO, HUVEC membrane integrity was measured for HUVECs plunged into liquid nitrogen without any controlled cooling.

### Two-Step Freezing

Aliquots of 200 μL of HUVEC suspensions in EGM in the absence of cryoprotectants were transferred to 6 × 50 mm glass culture tubes (VWR) and were rapidly cooled to an intermediate sub-zero hold temperature, which was between −3 °C and −40 °C. To prepare HUVEC suspensions in EGM in the presence of cryoprotectants, 100 μL of a 2X-concentrated cryoprotectant solution was mixed with 100 μL of HUVEC suspension. DMSO (Fisher Scientific, Edmonton, AB, Canada) was used as a permeating cryoprotectant and HES (molecular weight range from 200 to 300 kDa, Bristol-Myers Squibb, Dublin, Ireland) was used as a non-permeating cryoprotectant. The intermediate sub-zero hold temperature range was −5 °C to −40 °C when using 3–10% DMSO in the presence or absence of HES, and −10 °C to −45 °C when using 10% DMSO plus HES or when using 20% DMSO. After a two-minute thermal equilibration time, ice nucleation was induced using liquid nitrogen-cooled forceps and a three-minute hold time was allowed for latent heat removal and cell dehydration. HUVEC suspensions were either thawed directly from intermediate sub-zero hold temperatures (direct thaw) or plunged from an intermediate sub-zero hold temperature into liquid nitrogen, stored in liquid nitrogen for at least one hour, and then thawed (plunge thaw). All thawing steps were performed using a 37 °C water bath until the last sliver of ice had melted. After thawing, cells were left at room temperature for immediate membrane integrity assessment.

### Graded Freezing

Aliquots of 200 μL of HUVECs in the presence or absence of cryoprotectant were transferred to culture tubes. To prepare HUVEC suspensions in EGM in the presence of cryoprotectants, a 2X-concentrated cryoprotectant solution was mixed with an equal volume of HUVEC suspension. HUVECs in culture tubes were cooled from 0 °C to the first experimental temperature. The experimental temperature range was −3 °C to −40 °C in the absence of cryoprotectant, −5 °C to −40 °C when using 3–10% DMSO in the presence or absence of HES, and −10 °C to −45 °C when using 10% DMSO plus HES or when using 20% DMSO. After a two-minute thermal equilibration time at the first experimental temperature, ice nucleation was induced using liquid nitrogen-cooled forceps and three minutes was allowed for latent heat removal and cell dehydration prior to beginning the cooling at 1 °C/min or 0.2 °C/min. HUVEC suspensions were either thawed directly from experimental temperatures (direct thaw) or plunged from experimental temperatures into liquid nitrogen, stored in liquid nitrogen for at least one hour and then thawed (plunge thaw). All thawing steps were performed using a 37 °C water bath until the last crystal of ice had melted. After thawing, cells were left at room temperature for immediate membrane integrity assessment.

### Tube Formation Assay

Matrigel matrix basement membrane (Corning, Bedford, MA, USA) was thawed from –20 °C by leaving it overnight at 4 °C and keeping it on ice until use. A 289 μL aliquot was dispensed into each well of a chilled 24-well culture plate using pre-cooled pipette tips. The plate was incubated at 37 °C for 30–60 minutes to allow the Matrigel to solidify. In the meantime, fresh HUVECs in EGM were prepared for plating. After thawing previously cryopreserved samples, the cryoprotectants were removed by serial dilution using phosphate-buffered saline with 2% FBS followed by centrifugation and aspiration of the supernatant. The cell pellets were resuspended in media, and 300 μL of the cell suspension (containing approximately 1.2 × 10^5 ^HUVECs) were added to each well containing Matrigel. The plate was incubated for 16–18 hours at 37 °C and 5% CO_2_. Tube formation was observed at 40X magnification using the Labovert phase contrast microscope (Leitz, Los Angeles, CA, USA) and images were captured with an attached Diractor camera (Pixera, Santa Clara, CA, USA). ImageJ with Angiogenesis Analyzer plugin software was used to quantify the extent of tube formation^82^. The settings of the Angiogenesis Analyzer plugin are described in our previous work^79^.

### Statistics

Using the Student’s *t* distribution, two-tailed p-values that were less than 0.05 were considered to indicate significantly different population means.

## Additional Information

**How to cite this article**: Sultani, A. B. *et al*. Improved Cryopreservation of Human Umbilical Vein Endothelial Cells: A Systematic Approach. *Sci. Rep.* **6**, 34393; doi: 10.1038/srep34393 (2016).

## Supplementary Material

Supplementary Information

## Figures and Tables

**Table 1:** Membrane integrity of HUVEC controls in the presence and absence of DMSO.

Measured membrane integrity	In the absence of cryoprotectant	10% DMSO, 15 minute exposure	10% DMSO, 30 minute exposure	10% DMSO, multi-step exposure^15^	20% DMSO, 15 minute exposure
Pre-experiment	92.9 ± 0.7%	93.3 ± 0.7%	91.4 ± 1.3%	93.0 ± 0.5%	84.6 ± 0.4%
After 1 hour at 0 °C	90.6 ± 1.2%	91.7 ± 1.5%	90.5 ± 0.9%	91.0 ± 2.2%	77.1 ± 2.4%
Plunge from 0 °C into liquid nitrogen	0.07 ± 0.03%	0.8 ± 0.3%	2.1 ± 0.7%	4.6 ± 0.3%	2.0 ± 0.2%

The membrane integrity is expressed as mean ± standard error of the mean, N = 3.

**Table 2:** Maximum membrane integrities of HUVECs after incubation with various cryoprotectants for 15 minutes on ice, cooling to the nucleation temperature, nucleating ice, holding for 3 minutes and then cooling at 1 °C/min to various sub-zero temperatures, plunging into liquid nitrogen and then thawing.

Cryoprotectant(s)	Plunge temperature (°C)	Maximum observed membrane integrity (%)	Membrane integrity normalized to fresh control
10% DMSO	−35	67.4 ± 1.9	72.6 ± 1.8
20% DMSO	−40	59.2 ± 2.2	64.3 ± 1.6
10% DMSO + 10% HES	−45	78.7 ± 0.5	87.8 ± 2.0
10% DMSO + 8% HES	−45	83.6 ± 1.6	91.2 ±1.3
10% DMSO + 5% HES	−45	78.8 ± 0.9	83.6 ± 1.0
7% DMSO + 7% HES	−45	81.2 ± 2.2	87.4 ±2.2
7% DMSO + 6% HES	−45	77.7 ± 0.8	83.2 ± 0.5
5% DMSO + 6% HES	−35	87.7 ± 0.8	94.0 ± 0.9
3% DMSO + 6% HES	−35	79.3 ± 2.1	85.2 ± 2.0
Commercially-supplied		64.8 ± 2.2	

In the last column, the % membrane integrities were normalized relative to those of fresh, unfrozen (control) cells. The values are expressed as mean ± standard error of the mean; N = 3, except for commercially supplied HUVECs (N = 6).
