# Supplementary material for: Improved Cryopreservation of Human Umbilical Vein Endothelial Cells: A Systematic Approach
Source: Sci Rep. 2016 Oct 6;6:34393. doi: 10.1038/srep34393 (PMC5052637; doi:10.1038/srep34393)
Supplement: Supplementary Information [file srep34393-s1.pdf]

Supplementary Information for "Improved Cryopreservation of Human Umbilical Vein Endothelial Cells: A Systematic Approach"

A. Billal Sultani[a,b], Leah A. Marquez-Curtis[a,b], Janet A. W. Elliott*[,a,b], Locksley E. McGann[b]

[a] Department of Chemical and Materials Engineering, University of Alberta, Edmonton, Alberta, Canada

[b] Department of Laboratory Medicine and Pathology, University of Alberta, Edmonton, Alberta, Canada

* Corresponding author:
Janet A. W. Elliott
Department of Chemical and Materials Engineering
Donadeo Innovation Centre for Engineering
University of Alberta, Edmonton, AB Canada T6G 1H9
Phone: (780)-492-7963
Fax: (780)-492-2881
Email: janet.elliott@ualberta.ca

Figure S1 shows flow cytometry measurement of membrane integrity for a mixture of membrane-intact and membrane-damaged HUVECs using two fluorescent stains (SYTOEB and SYTOPI) prepared as described in the Methods section of the manuscript.

A mixture of membrane-intact and membrane-damaged HUVECs was used to identify HUVEC populations for counting. Membrane-intact HUVECs were prepared from fresh HUVECs. Membrane-damaged HUVECs were prepared from HUVECs plunged into liquid nitrogen directly from 0 °C. The gridlines used to identify the quadrants were defined using histograms of green and red intensity. The grid center was determined from: *i)* the minimum between the background and High green domain, and *ii)* the minimum between the background and High red domain. The colour of cells from the quadrants was conserved for display on forward scatter versus side scatter plots. The histograms for high green and high red detection, fluorescence measurements after compensation and forward scatter (FS) and side scatter (SS) of events are shown.

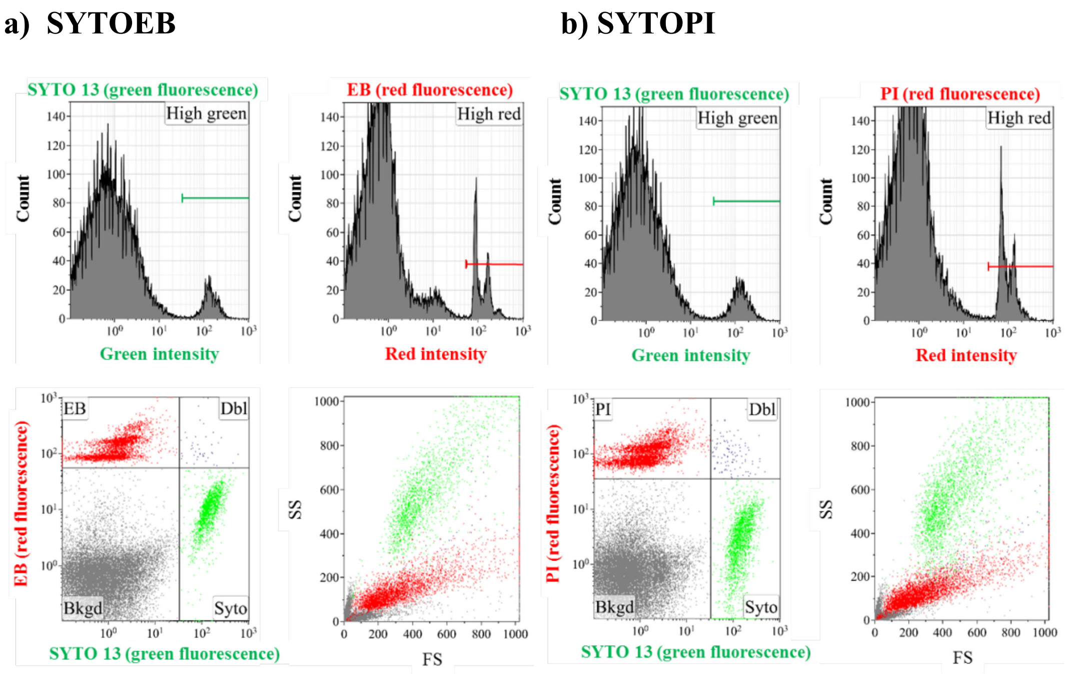

Figure S1. Flow cytometry measurement of membrane integrity for a mixture of membrane-intact and membrane-damaged HUVECs using (a) SYTOEB or (b) SYTOPI. The histograms for high green and high red detection, fluorescence measurements after compensation and forward scatter (FS) and side scatter (SS) of events are shown. Fluorescence measurements in the SYTO quadrant (green events) corresponded to membrane-intact cells, fluorescence measurements in the EB or PI quadrant (red events) corresponded to membrane-damaged cells, and fluorescence measurements in the Dbl quadrant (blue events) were included in membrane-damaged cell counts. Which of these stains (SYTOEB or SYTOPI) was used in each experimental run is detailed in a thesis[1], and the number of repeats with each stain is summarized in Supplementary Table S1.

Double peaks were observed from the red fluorescence of EB or PI. One peak was approximately twice the red fluorescence intensity of the other peak. The more intense red fluorescence peak could be the result of a clump of two cells or cells undergoing DNA synthesis, while the less intense red fluorescence peak could be the result of single cells in interphase of the cell cycle[2]. In the presence of EB, a small third peak which has approximately twice the red fluorescence intensity could be the result of clumps of four cells or clumps of two cells undergoing DNA synthesis. It was not the intent of this work to analyze HUVEC cycling, but for cell-cycle analysis, the red fluorescence peak area and peak height may be important to discriminate cell clumps[2].

Supplementary Table S1. Membrane integrity staining for each interrupted cooling experiment

	Number of membrane integrity staining experiments (N)	
Interrupted cooling protocol	SYTOEB	SYTOPI
No cryoprotectant		
Two-step freezing	3	0
Graded freezing, 0.2°C/min	3	0
Graded freezing, 1°C/min	2	1
10% DMSO		
Graded freezing, 1°C/min, (15 minute exposure)	3	0
Graded freezing, 1°C/min, (30 minute exposure)	3	0
Graded freezing, 1°C/min, (10/20 minute exposure)	3	0
Graded freezing, 0.2°C/min	1	2
Two-step freezing	1	2
Graded freezing, 1°C/min, 20% DMSO	0	3
Graded freezing, 1°C/min, 10% DMSO + 5% HES	0	3
Graded freezing, 1°C/min, 10% DMSO + 8% HES	0	3
Graded freezing, 1°C/min, 10% DMSO + 10% HES	0	3
Graded freezing, 1°C/min, 7% DMSO + 7% HES	0	3
Graded freezing, 1°C/min, 7% DMSO + 6% HES	0	3
Graded freezing, 1°C/min, 5% DMSO + 6% HES	0	3
Graded freezing, 1°C/min, 3% DMSO + 6% HES	0	3

References

1. Sultani, A. B. Protocol designs to optimize cryopreservation of human umbilical vein endothelial cells. MSc thesis. (2015).

2. Wersto, R. P. *et al.* Doublet discrimination in DNA cell-cycle analysis. *Cytometry* **46,** 296–306 (2001).
